# Predictors of outcomes in patients with repeat surgery for obstetric fistula: a retrospective review

**DOI:** 10.1007/s00192-022-05421-0

**Published:** 2023-01-06

**Authors:** Lennart P. Maljaars, Helai Hesham, Hiske Huisman, William Nundwe, Jan-Paul W. R. Roovers, Rachel J. Pope

**Affiliations:** 1 Department of Obstetrics and Gynecology, Amsterdam UMC, Amsterdam Medical Center, Room H4-240, Meibergdreef 9, 1105 AZ Amsterdam, the Netherlands; 2 Amsterdam Reproduction and Development Research Institute, Amsterdam, The Netherlands; 3 Division of Female Pelvic Medicine and Reconstructive Surgery, Department of Obstetrics and Gynecology, Columbia University Medical Center, New York, USA; 4 Baylor Malawi Foundation, Lilongwe, Malawi; 5 Division of Female Sexual Health, Urology Institute, University Hospitals Cleveland Medical Center, Cleveland, OH USA

**Keywords:** Fistula closure, Obstetric fistula, Repeat surgery, Residual incontinence, Surgical outcomes, Vesicovaginal fistula

## Abstract

**Introduction and hypothesis:**

Predictors of surgical outcomes in patients with an obstetric fistula who have been operated before should be identified in order to guide surgical strategy and optimize counseling of the patient.

**Methods:**

This retrospective study is aimed at identifying predictors of outcomes for repeat surgery in 346 patients who had been operated on before for an obstetrics fistula at the Fistula Care Center (FCC) in Lilongwe, Malawi. Repeat cases were only undertaken by advanced and expert surgeons. The primary outcome was successful anatomical closure, based on a negative postoperative dye test. The secondary outcomes involved urinary continence, based on a patient-reported questionnaire and an objective 1-h pad weight test. Logistic regression models were used to test the predictors for statistical significance.

**Results:**

Successful fistula closure was achieved in 288 (83%) patients and continence was achieved in 185 (64%) patients after the first repeat attempt at the FCC. Lack of urethral involvement (Goh classification: proximity to the urethra) was shown to be a good predictor of the outcomes: fistula closure and subjective and objective continence.

**Conclusions:**

Absence of urethral involvement is an independent predictor for successful outcomes in repeat surgery for obstetric fistulas. Even in the hands of an expert surgeon, the risk of another failure in achieving anatomical closure or subjective or objective continence is between 4 and 5 times higher than when the urethra is not involved.

**Supplementary information:**

The online version contains supplementary material available at 10.1007/s00192-022-05421-0

## Introduction

Obstetric fistulas are a type of birth-related trauma to the pelvic floor that results in continuous leakage of urine or feces through the vagina and severely affect the physical, mental, social, and economic wellbeing of patients [1, 2]. Around 2–3 million women worldwide are suffering from an obstetric fistula, with an estimated 50,000–100,000 new cases each year [3–6]. Most of these women live in sub-Saharan Africa and Asia with limited access to obstetric and gynecological care [7, 8]. Although fistula prevention through adequate antenatal care and access to obstetric emergency facilities remains the most important long-term global strategy, surgical repair is the only treatment for the gross majority of affected women [5, 6]. However, not all attempted surgical repairs lead to the clinical successes of both fistula closure and continence. Even when anatomical closure is achieved, patients may still suffer from residual incontinence as a consequence of destruction of the continence mechanism; described as the ‘continence gap’ [9]. These women often seek care again and may be indicated for repeat surgery intending fistula closure and/or continence for urine and stool. Subsequent attempts are often less successful than the original attempt, likely because of the formation of scar tissue, loss of vascularity, and loss of natural tissue planes, making surgery even more challenging [10, 11]. In obstetric fistula repair, the literature shows that fistula closure rates are negatively affected by small bladder size, prior repairs, severe vaginal scarring, and partial or extensive involvement of the urethra. The same factors, except bladder size, also predict continence [10, 12, 13]. Multiple studies failed to show the independent predictive value of patient-related factors and little is known about context-related factors [12]. To date, little research has addressed which determinants play a role in predicting surgical outcomes of repeat attempts. Gaining more insight into patient-, fistula- and context-related predictors for surgical success in first and subsequent repairs is of the utmost importance to guide future surgical strategy and patient counseling on expected treatment outcomes. This way, a patient can be better counseled if complete cure is achievable and if a subsequent attempt is going to increase the chances of cure or that another surgical intervention should be avoided. Therefore, the objective of this study is to identify predictors of successful fistula closure and continence of repeat fistula repair surgery.

## Materials and methods

### Study design

This is a retrospective review of patients who received repeated fistula surgery in the Fistula Care Center (FCC) in Lilongwe, Malawi. This is part of a larger study on the outcomes of the surgical repair of obstetric fistula, which is approved by Baylor College of Medicine and the Malawian National Health Science Committee on Research (#929). Written informed consent was provided by all patients.

### Sample/study population (inclusion and exclusion criteria)

Patients who visited the FCC between 2010 and 2019 were screened for eligibility. Patients who had a previous fistula repair at an outside hospital, as well as those who had previous surgery at the FCC were included in the cohort. Repeat surgery was defined as women receiving a second or subsequent surgical intervention for their obstetric fistula. Patients who required multiple staged surgeries (with removal of bladder stones, staged rectovaginal fistula closure, etc.) or who had a non-obstetric fistula (e.g., malignant disease, sexual trauma, pelvic surgery) were excluded from the study. Patients with a missing primary outcome were also excluded from the analysis.

### Surgical technique

All repeat cases were performed by an advanced or expert surgeon at the FCC; less experienced surgeons did not perform repeat cases. Patients received different surgical procedures varying from vesicovaginal fistula repair, circumferential urethral repair, the use of different flaps (e.g., Singapore fasciocutaneous flap or gracilis muscle flap), sling procedures for incontinence and urinary diversion procedures as the last resort to achieve continence. The preferred approach was transvaginal in lithotomy position, with a small portion of the procedures carried out through an abdominal approach. Spinal anesthesia was preferred when possible. Patients received prophylactic antibiotics before surgery. Postoperatively, patients received vaginal packing the first 24 h after surgery and an indwelling catheter for a duration determined by the surgeon.

### Outcome measures

The primary outcome was successful anatomical closure of the fistula, based on a negative postoperative dye test. The test was performed in the hospital by a clinician, after a period of bladder catheterization. The test was scored negative if no leaks were identified and positive if a leak was identified. The secondary outcome was urinary continence, based on a patient-reported continence grade. The continence grade is a subjective grading system with the following answer options: “cured, no incontinence,” “incontinence with cough strain or exertion,” “incontinence on walking,” “incontinence on walking, sitting, and/or lying but still voiding some urine,” and “incontinence on walking, sitting, and/or lying but not voiding any urine” [14]. The patient was scored as ‘continent’ when a patient reported “cured, no incontinence.” In all other instances the patient was scored ‘incontinent.’ A pad weight test was also performed to quantify urinary incontinence objectively [15]. Patients received a pre-weighted pad after catheter removal and were asked to continue their daily activities for 60 min. After an hour the pad was weighed again and the difference was calculated. A pad weight of <1.5 g was defined as negative or ‘continent’ [16]. Known predictors from the literature (previous surgery, urethral involvement, fistula size and vaginal scarring (Goh classification)) were included in the logistic regression model [10–12, 17]. The Goh classification is a classification tool for female genital tract fistula and scores injuries based on the distance to the external urethral opening, the size of the fistula and associated scarring, vaginal length, or special considerations [17].

### Data collection

Data collection was performed by trained research personnel using RedCap (Research Electronic Data Capture, Vanderbilt University) and checked by a medical research student and a physician. The following patient characteristics were collected from patient charts: age, BMI, HIV status, history of fistula repairs, type of injury based on the Goh classification, type of surgery, surgical duration, and estimated blood loss.

### Statistical analysis

Analysis was performed with the use of IBM SPSS Statistics for Windows, version 28.0 (IBM Corp.). All data were assessed for normality. Normally distributed data were reported as mean and standard deviation, non-normally distributed data were reported as median with 25th and 75th percentiles, and categorical data were reported as numbers and percentages. Single imputation based on all predictor and outcome variables in the model was used to correct for the missing entries. To assess predictors of interest, logistic regression was used for the primary and secondary outcomes. *p* values less than 0.05 were considered statistically significant.

## Results

### Study population

Between 2010 and 2019, a total of 2,366 procedures were performed at the Fistula Care Center (FCC) in Lilongwe, Malawi. The flowchart of the included and excluded procedures at the FCC is shown in Supplementary Fig. 1. A total of 346 patients had complete data of the primary outcome on their first repeat attempt at the FCC and were included in the cohort. Seventy-two (20.8%) patients only received surgical repair at the FCC and 274 (79.2%) patients had already undergone surgical repair at another facility.

### Missing data

For 26 patients (7.5%) there were missing data on one of the predictor variables that were used in the logistic regression models. The data were missing at random owing to incomplete entries in the database. The percentages of missing data for the Goh classification (proximity to the urethra, size of the fistula, and special considerations) were 9.4%, 9.3%, and 9.4% respectively.

### Baseline characteristics

The baseline characteristics of the included women at time of enrolment at the FCC are shown in Table 1. The mean age of the included women was 35.0 [28–45] years old. The surgical details of the first repeat attempt can also be found in Table 2. The initial injury based on the Goh classification was mostly type 3 fistula (34.4%), followed by type 1 (28.3%), type 2 (21.4%), and type 4 (15.9%) fistulas. Most fistulas were small (60.1%) and special circumstances (e.g. ureteric involvement, circumferential fistula; 64.5%) were present in most cases. Most patients received vesicovaginal fistula closure (74.2%) at their first repeat attempt, 43 (12.5%) patients received urethrovaginal fistula closure, 42 (12.2%) received circumferential urethral defect repair, and 6 (1.7%) patients received an end-stage urinary diversion procedure during their first repeat attempt at the FCC. The median surgery time was 1:00 [0:40–1:25] h with an estimated blood loss of 50.0 [20–100] ml. The median duration of postoperative catheterization was 14 [14–20] days.

**Table 1:** Baseline patient characteristics

Patient characteristics	*n*		
Age (years)^a^	341	35.0	(28–45)
BMI^a^	316	22.4	(20–25)
HIV status^b^	343		
Negative Positive		316	(92.1)
		27	(7.9)
History of previous repair at another facility^b^	346		
Yes		274	(79.2)
No		72	(20.8)
Number of previous repairs^b^	346	1.0	(1)
Surgical approach in previous surgery^b^	274		
Abdominal		45	(16.4)
Vaginal		208	(75.9)
Abdominal and vaginal		17	(6.2)
Unknown		4	(1.5)

*BMI* body mass index, *HIV* human immunodeficiency virus

^a^Median (25th to 75th percentile)

^b^Number of patients (percentage)

**Table 2:** Surgical characteristics

Surgical characteristic	*n*		
Initial injury based on Goh classification^a^			
Proximity to the urethra	346		
Type 1: distal edge of fistula >3.5 cm from external urethral meatus		98	(28.3)
Type 2: distal edge 2.5–3.5 cm from external urethral meatus		74	(21.4)
Type 3: distal edge 1.5–2.5 cm from external urethral meatus		119	(34.4)
Type 4: distal edge <1.5 cm from external urethral meatus		55	(15.9)
Size of fistula	346		
A: <1.5cm		212	(60.1)
B: 1.5–3cm		88	(25.4)
C: >3cm		46	(13.3)
Special considerations	346		
I: none or mild fibrosis; vaginal length >6cm, normal bladder capacity;		69	(19.9)
II: moderate or severe fibrosis and/or marked reduction in vaginal length and/or bladder capacity		54	(15.6)
III: special circumstances^b^		223	(64.5)
Procedures^c^	346		
Vesicovaginal fistula repair		256	(74.2)
Urethrovaginal fistula repair		43	(12.5)
Concomitant rectovaginal fistula repair		8	(2.3)
Circumferential urethral defect repair		42	(12.2)
Urethroplasty (urethral plication)		27	(7.8)
Refixation of pubococcygeal fascia		25	(7.2)
Ureteric reimplantation		13	(3.8)
Urinary diversion		6	(1.7)
Anesthesia^c^	344		
Spinal		331	(96.2)
General		13	(3.8)
Surgical duration^c^ (min)	335	1:00	(0:40–1:25)
Estimated blood loss^c^ (ml)	323	50.0	(20–100)
Postoperative catheter duration^c^ (days)	100	14.0	(14–20)

^a^Number of patients (percentage)

^b^For example, ureteric involvement, circumferential fistula

^c^Median (25th to 75th percentile)

### Outcomes

**Fig. 1:**
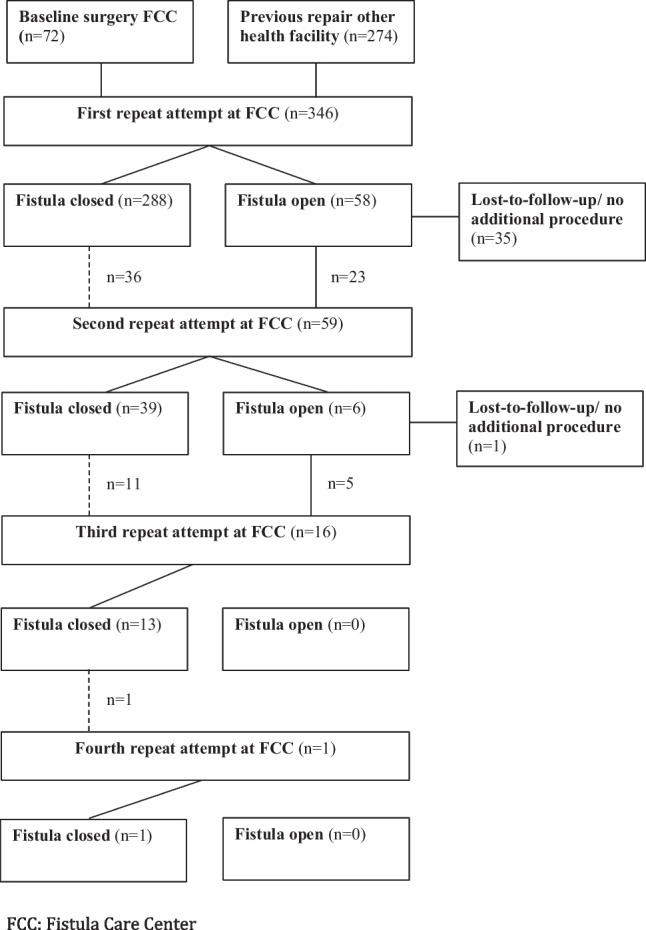
Flowchart of the repeat attempts at Fistula Care Center (*FCC*). Number of patients and repeat attempts. The *interrupted line* shows the patients who needed an additional attempt, despite having a negative postoperative dye test (primary outcome)

After the first repeat attempt, 288 patients (83.2%) achieved fistula closure, based on a negative dye test. In 58 patients (16.8%), the fistula remained open. Some patients with a negative dye test still required subsequent attempts. After the fourth (final) repeat attempt, all of the included patients achieved full anatomical closure. The detailed flowchart including all subsequent repeat attempts at the FCC are shown in Fig. 1. Subjective continence was present in 185 patients (64%), with 104 patients (36%) still have complaints of some form of urinary incontinence. Based on the pad weight test <1.5 g, 146 patients (42.4%) were continent and 152 patients (43.9%) had a positive pad weight test after catheter removal.

Logistic regression was used to assess predictors for all three outcomes: fistula closure and subjective and objective continence. The surgical history at another facility, number of previous attempts and Goh classification were added to the model to predict the effect of these independent variables on the different outcomes. The results are shown in Table 3. The proximity of the fistula edge to the urethra was a predictor for all three outcomes: patients with a fistula with urethral involvement were 4–13 times less likely to have fistula closure (type 2 OR=0.19, 95% CI [0.06–0.60]; type 3 OR=0.26, 95% CI [0.08–0.83]; type 4 OR=0.08, 95% CI [0.02–0.25]), 4–8 times less likely to report subjective continence (type 3 OR=0.24, 95% CI [0.12–0.50]; type 4 OR=0.12, 95% CI [0.05–0.30]) and 4–6 times less likely to have a pad weight <1.5 g (type 2 OR=0.27, 95% CI [0.13–0.55]; type 3 OR=0.20, 95% CI [0.11–0.39]; type 4 OR=0.17, 95% CI [0.07–0.40]). The only exception was a type 2 fistula that did not predict subjective continence (*p*=0.075). A patient with a fistula size > 3 cm was 3 times more likely to have residual incontinence based on the subjective continence grade (OR=0.32, 95% CI [0.14–0.74], *p*=0.008). Based on our data, the number of previous attempts and surgical history at another facility had no predictive value for any of the outcomes.

**Table 3:** Predictors for surgical outcome of repeat attempt fistula surgery

Primary outcome: closure based on negative dye test (*n*=346)	OR (95% CI)	*p* value
Surgical history at another facility	0.90 (0.41–2.00)	0.801
Number of previous attempts	0.83 (0.58–1.19)	0.307
Proximity to the urethra		
Type 1: distal edge of the fistula >3.5 cm from the external urethral meatus	Reference	
Type 2: distal edge 2.5–3.5 cm from the external urethral meatus	0.19 (0.06–0.60)	0.005
Type 3: distal edge 1.5–2.5 cm from the external urethral meatus	0.26 (0.08–0.83)	0.022
Type 4: distal edge <1.5 cm from the external urethral meatus	0.08 (0.02–0.25)	<0.001
Size of fistula		
A: <1.5 cm	Reference	
B: 1.5–3 cm	0.53 (0.27–1.04)	0.066
C: >3 cm	0.52 (0.22–1.24)	0.139
Special considerations		
I: none or mild fibrosis; vaginal length >6 cm, normal bladder capacity	Reference	
II: moderate or severe fibrosis and/or marked reduction in vaginal length and/or bladder capacity	0.36 (0.10–1.27)	0.112
III: special circumstances^a^	0.50 (0.16–1.57)	0.236
Secondary outcome: continence based on continence grade (*n*=289)		
Surgical history at another facility	0.62 (0.29–1.34)	0.224
Number of previous attempts	0.90 (0.63–1.30)	0.585
Proximity to the urethra		
Type 1: distal edge of fistula >3.5 cm from the external urethral meatus	Reference	
Type 2: distal edge 2.5–3.5 cm from the external urethral meatus	0.47 (0.21–1.01)	0.075
Type 3: distal edge 1.5–2.5 cm from the external urethral meatus	0.24 (0.12–0.50)	<0.001
Type 4: distal edge <1.5 cm from the external urethral meatus	0.12 (0.05–0.30)	<0.001
Size of the fistula		
A: <1.5 cm	Reference	
B: 1.5–3 cm	0.74 (0.40–1.37)	0.331
C: >3 cm	0.32 (0.14–0.74)	0.008
Special considerations		
I: none or mild fibrosis; vaginal length >6 cm, normal bladder capacity	Reference	
II: moderate or severe fibrosis and/or marked reduction in vaginal length and/or bladder capacity	0.40 (0.15–1.05)	0.062
III: special circumstances^a^	0.44 (0.20–0.97)	0.041
Secondary outcome: continence based on pad weight test (*n*=298)		
Surgical history at another facility	0.74 (0.38–1.42)	0.363
Number of previous attempts	0.76 (0.52–1.12)	0.166
Proximity to the urethra		
Type 1: distal edge of the fistula >3.5 cm from the external urethral meatus	Reference	
Type 2: distal edge 2.5–3.5 cm from the external urethral meatus	0.27 (0.13–0.55)	<0.001
Type 3: distal edge 1.5–2.5 cm from the external urethral meatus	0.20 (0.11–0.39)	<0.001
Type 4: distal edge <1.5 cm from the external urethral meatus	0.17 (0.07–0.40)	<0.001
Size of fistula		
A: <1.5 cm	Reference	
B: 1.5–3 cm	0.93 (0.52–1.67)	0.802
C: >3 cm	1.02 (0.48–2.21)	0.951
Special considerations		
I: none or mild fibrosis; vaginal length >6 cm, normal bladder capacity	Reference	
II: moderate or severe fibrosis and/or marked reduction in vaginal length and/or bladder capacity	0.86 (0.37–2.00)	0.732
III: special circumstances^a^	0.67 (0.34–1.31)	0.239

^a^For example, ureteric involvement, circumferential fistula

*OR* odds ratio, *CI* confidence interval

## Discussion

In our study, fistula closure was achieved in 83% of patients undergoing their first re-operation for obstetric fistula at the FCC. Continence, based on the subjective continence grade, was realized in about 2 out of 3 patients. Logistic regression models showed that the distance between the fistula edge and the external urethral meatus appeared to be an independent risk factor for all relevant surgical outcomes, with the exception of a type 2 fistula, which had no significant predictive value for subjective continence. In addition, a fistula size of more than 3 cm appeared to predict subjective continence. A longer distance between the fistula and the external meatus of the urethra, and therefore less involvement of the urethra itself, has a better chance of surgical success. When the urethra is damaged, surgical re-anastomosis of the urethra or creation of a neourethra can restore the anatomy. However, this does not necessarily result in functional recovery as the sphincter-closing mechanism may be destroyed. These findings are in line with existing literature [10, 12, 13].

We were able to demonstrate one factor with predictive value for successful surgical outcome. In another study on patients with recurrent fistulas, significant associations were demonstrated between the number of previous repairs, age, fistula classification, duration of the fistula, surgeon’s experience, and the place where the repair was attempted [18]. However, no multivariate analysis was performed to control for confounders and probably most significance would be lost if a multivariate analysis were used, as shown in our study. In contrast to studies on first attempts at obstetric fistula repair, the predictive value of surgical history and vaginal scarring could not be confirmed in our study evaluating repeat surgery [10, 12, 13]. We were unable to assess patient-related characteristics as the sample size was too small to add more variables in the multivariate regression analysis. Therefore, patient-related predictors were not included. There were no context-related factors with predictive value in repeat cases; the location of the first surgery and the number of previous surgeries did not affect the outcomes.

The center where our patients underwent surgery serves as a regional expertise center for fistula care and all repeat cases were performed by experienced surgeons. Our study shows that despite the experience of the surgeons, the likelihood of another surgical failure in repeat cases is high, especially when it comes to realizing urinary continence. This warrants a cautious approach and counseling. Patients should be counseled optimally to facilitate shared decision-taking. In low-resource settings in particular, patients may be inclined to take any opportunity to receive care and the surgeon’s opinion may be directive and decisive.

The overview of the surgical journey of obstetric fistula patients shown in this study highlights the discrepancy between anatomical closure and continence. Women may experience residual incontinence despite anatomical closure of the fistula, a phenomenon known as ‘the continence gap’ [9]. In addition, this study shows that the majority of patients who needed a second, third, or fourth attempt had a negative postoperative dye test, which should indicate cure (Fig. 1). These discrepancies show that anatomical closure may not be the best outcome to define success and predict the need for additional surgeries. Subjective or objective continence may be a better predictor of successful treatment. Ideally, a composite outcome for obstetric fistula repair would incorporate anatomical closure and subjective and objective urinary continence to determine surgical success and optimize indication for surgery.

This study was performed to better inform patients with a failed first attempt about their chance of surgical success. A strength of this study was that our multivariate analysis was performed in a cohort large enough to estimate several fistula- and context-related predictors without overfitting [19]. In addition, the period from which patients were included was long enough to visualize the success rate after multiple attempts. Finally, three different outcomes were included in the study to assess the predictive value of these outcomes and to assess both objective and subjective definitions of outcome.

One of the limitations of our study was that the cohort was not big enough to include more potential prognostic variables. Another limitation is that our data do not allow us to determine the cause of incontinence in each individual patient. The subjective continence grade contains the category “incontinence with cough strain or exertion,” indicating stress urinary incontinence, but there was no objective measurement, such as urodynamic investigation, to confirm this. A postoperative cough stress test could also help to assess the type of incontinence, but was not routinely assessed in our study. More insight into the exact type of urinary incontinence would optimize the counseling of patients.

## Conclusion

In conclusion, the absence of urethral involvement (type 1 fistula) is an independent predictor of successful outcomes of repeat obstetric fistula repair when performed by a specialized fistula surgeon. The lack of other prognostic factors for surgical success makes it challenging to optimally counsel patients who consider repeat surgery.

## Supplementary information

ESM 1 (PDF 212 kb)
