# Supplementary figures and images for: Predictors of outcomes in patients with repeat surgery for obstetric fistula: a retrospective review
Source: Int Urogynecol J. 2023 Jan 6;34(7):1567–74. doi: 10.1007/s00192-022-05421-0 (PMC10287811; doi:10.1007/s00192-022-05421-0)

## Supplementary figure 1 – Flowchart patients FCC

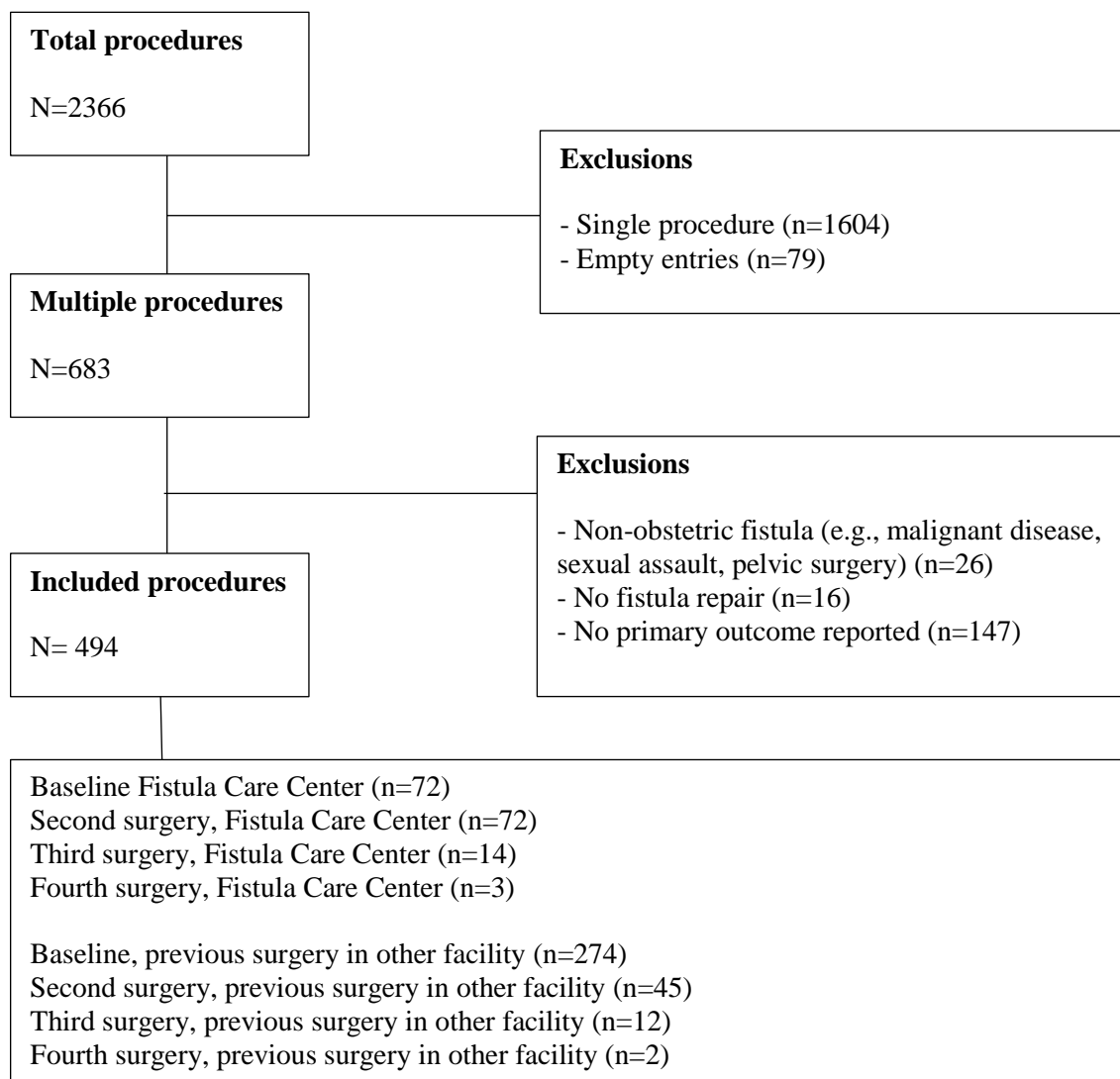

Supplement: Supplementary file 1 — (PDF 212 kb) [file 192_2022_5421_MOESM1_ESM.pdf]
